# Effects of Elevated Ambient Temperature on Reproductive Outcomes and Offspring Growth Depend on Exposure Time

**DOI:** 10.1100/2012/359134

**Published:** 2012-07-31

**Authors:** Huda Yahia Hamid, Md Zuki Abu Bakar Zakaria, Goh Yong Meng, Abd. Wahid Haron, Noordin Mohamed Mustapha

**Affiliations:** ^1^Department of Anatomy, Faculty of Veterinary Medicine, University of Khartoum, Khartoum 13314, Sudan; ^2^Department of Veterinary Preclinical Sciences, Faculty of Veterinary Medicine, Universiti Putra Malaysia, Selangor, 43400 Serdang, Malaysia; ^3^Department of Veterinary Clinical Studies, Faculty of Veterinary Medicine, Universiti Putra Malaysia, Selangor, 43400 Serdang, Malaysia; ^4^Department of Veterinary Pathology & Microbiology, Faculty of Veterinary Medicine, Universiti Putra Malaysia, Selangor, 43400 Serdang, Malaysia

## Abstract

Reproductive performance has been shown to be greatly affected by changes in environmental factors, such as temperature. However, it is also crucial to identify the particular stage of pregnancy that is most adversely affected by elevated ambient temperature. The aims of this study were to determine the effect on reproductive outcomes of exposure to elevated ambient temperature during different stages of pregnancy and to determine the effect of prenatal heat stress on offspring growth. Sixty pregnant rats were used in this study. The rats were divided equally into four groups as group 1 (control), group 2 (exposed to elevated temperature following implantation), group 3 (exposed to elevated temperature during pre- and periimplantation), and group 4 (exposed to elevated temperature during pre- and periimplantation and following implantation). Groups 3 and 4 had prolonged gestation periods, reduced litter sizes, and male-biased sex ratios. Moreover, the growth patterns of group 3 and 4 pups were adversely affected by prenatal exposure to elevated temperature. The differences between group 1 and group 3 and between group 1 and group 4 were highly significant. However, no significant differences were observed between groups 1 and 2 in the gestation length, sex ratios, and growth patterns. Thus, it can be concluded that exposure to elevated ambient temperature during pre- and periimplantation has stronger adverse effects on reproductive outcomes and offspring growth than postimplantation exposure.

## 1. Introduction

Reproductive performance has been shown to be greatly affected by changes in environmental factors, such as temperature. The most economically important reproductive performance traits of farm animals may be endangered by high environmental temperature [1]. In tropical countries and in summer of temperate countries, the reproductive performance of dairy cattle is low [2, 3]. Bloemhof et al. [1] observed that the pregnancy outcomes of sow decrease in the hot weather. Furthermore, the reproductive efficiency of Holstein cows is lower in autumn than in winter, suggesting a late effect of high temperature during the summer [4]. Both male and female reproductive processes are sensitive to high temperature. High environmental temperature has adverse effect on spermatogenesis and oocyte development and maturation [5, 6]. Fortunately, the reduced quality of the sperm and ova during the hot season has been overcome to some extent by artificial insemination (AI) and embryo transfer techniques [6]. However, overcoming the problems resulting from exposure to elevated ambient temperatures during pregnancy is more complicated. Ealy et al. [7] reported that exposure to elevated temperatures at day 1 of pregnancy in cow compromises early embryonic development. In mice, several studies have shown that exposure to elevated temperatures for 12 h soon after mating disturbs normal embryonic development [8–11]. On the other hand, exposure of pregnant ewes to high temperatures during midgestation causes reduced foetal weights [12].

The recent climate changes and global warming have led to renewed interest in the study of the effects of exposure to elevated ambient temperatures on pregnancy outcomes and offspring growth with special attention to the time of exposure that leads to more deleterious effects on pregnancy outcomes. Determining the pregnancy stage that is most sensitive to heat exposure will assist in the design of environmental modification systems that provide an optimal temperature at critical period of pregnancy to improve the pregnancy rates and outcomes in hot climates. Therefore, the current study was intended to determine the effects of exposure to elevated ambient temperature during different pregnancy stages on reproductive outcomes and offspring growth and to determine the stage of pregnancy that is most sensitive to elevated temperature.

## 2. Materials and Methods

### 2.1. Animals

Sixty virgin female Sprague Dawley rats were used in this study. These in-house bred rats were 2-3 months old and weighed 200–220 g. The animals were maintained under a controlled lighting regime of 12 hours light and 12 hours dark; the lights were turned on at 0700 h and off at 1900 h. The rats were maintained under a temperature of 23 ± 1°C and relative humidity of 55–65%. The rats were allowed free access to chow and water. The animal care and handling throughout the study were approved by the Animal Care and Use Committee of the Faculty of Veterinary Medicine, Universiti Putra Malaysia, Malaysia.

Only females that showed at least two consecutive, regular four-day estrous cycles immediately before the study were used. Proestrus females were caged overnight with a male from the same strain with an age of 4-5 months, a weight of 400–500 g, and proven fertility. The presence of spermatozoa in a vaginal smear performed the next morning was used to detect successful copulation, and that day was designated as day 1 of the pregnancy.

### 2.2. Experimental Design

Parental influence was controlled by mating each male with four littermate females. On pregnancy day 1, each rat was randomly assigned to one of the following groups.

In group 1 (control, *n* = 15), the rats were maintained under optimal temperature (23 ± 1°C) from pregnancy day 1 until parturition. 

In group 2 (*n* = 15), the rats were kept under optimal temperature (23 ± 1°C) during pre- and periimplantation, and, on day 8, they were transferred to a room without an air conditioner or fan and remained there until parturition. The temperature in the room was 33 ± 2°C, and the relative humidity was 80–86%.

In group 3 (*n* = 15), the rats were subjected to the elevated temperatures (33 ± 2°C) during pre- and periimplantation (from day 2 to day 7), and, on day 8, they were transferred to optimal conditions and remained there until parturition.

In group 4 (*n* = 15), the rats were maintained under elevated temperatures (33 ± 2°C) from day 2 of pregnancy until parturition.

**Figure 1:**
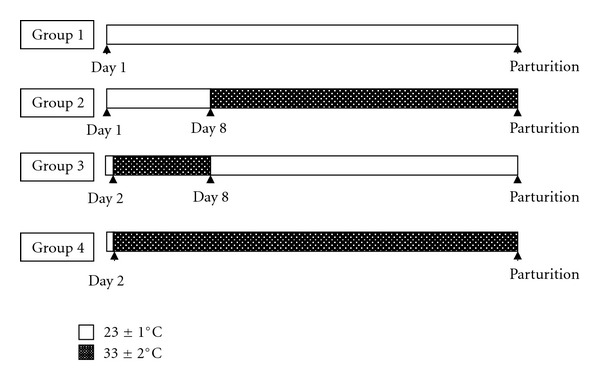
Schematic illustration of pregnancy period and time of exposure to elevated temperatures in the four groups.

After parturition, dams of groups 2 and 4 and their pups were returned to the room with optimal temperature (23 ± 1°C) and maintained there until the end of the study. The schematic illustration of experimental design is shown in Figure 1.

The length of gestation, litter sizes, neonatal deaths, sex ratios (male proportions), and body weights were recorded and compared among the four groups. 

The body weight was measured immediately after birth and at one week, two weeks, and three weeks. Litters (*n* = 6) were culled to eight pups, with sex distribution kept as equal as possible in each litter. The mean weight of the male and female pups of each dam was recorded separately.

### 2.3. Statistical Analysis

The gestational length, litter size, and sex ratio in the different groups were compared using a one-way analysis of variance (ANOVA). The weights of the four groups' pups were compared using repeated measures ANOVA. Tukey's test was used for post hoc analyses. The statistical analysis was carried out using the PASW statistics 18 package (SPSS Inc., Chicago, IL, USA) with the significance level at *P* < 0.05. All the data are presented as the mean ± SEM.

## 3. Results

### 3.1. Gestation Length and Time of Birth

**Figure 2:**
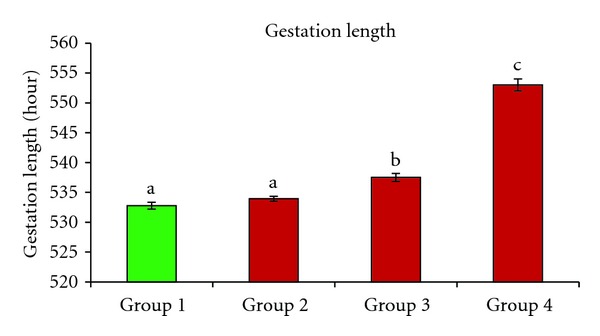
The gestation lengths of the control group (group 1) and groups exposed to elevated ambient temperature. The bars represent the mean ± SEM. Data labeled with different letters are significantly different at *P* < 0.001.

Gestation length and time of birth were adversely affected by exposure to elevated temperatures. The longest gestation length was recorded in group 4. There was a significant increase (*P* < 0.001) in the gestation length of group 4 when compared with that of the control group (Figure 2). No significant difference was found between group 2 and the control group (*P* > 0.05), while group 3 had a significantly longer gestation length than the control group (*P* = 0.001) and group 2 (*P* < 0.01).

All of the females in the control group gave birth on day 23 of pregnancy before 11:00 h. In group 2, 87% of the females gave birth on day 23 before 11:00 h and the rest (13%) gave birth from 12:00 h to 13:30 h. The group 3 females gave birth later on day 23 of pregnancy, with 80% of the births occurring from 15:00 h to 18:00 h and 20% of births occurring from 12:00 h to 14:00 h. In group 4, the births occurred on day 24 of pregnancy, with 93% of the females giving birth in the early morning and 7% giving birth from 11:00 h to 12:00 h.

### 3.2. Litter Size

**Figure 3:**
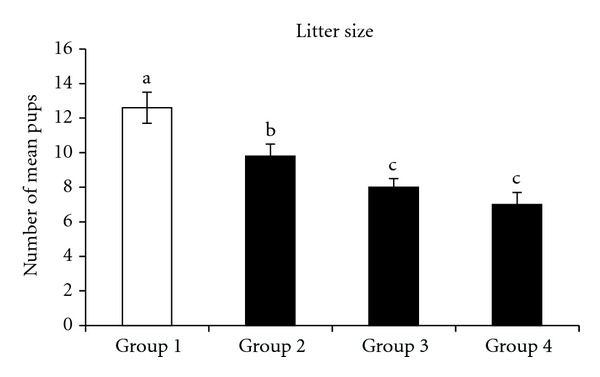
The litter sizes produced by the females of the control group (group 1) and groups exposed to elevated ambient temperature. The bars represent the mean ± SEM. Data labeled with different letters are significantly different at *P* < 0.05.

Results of litter sizes are shown in Figure 3. The largest litter size was recorded in the control group, while the smallest was found in group 4; the difference between the two was highly significant (*P* < 0.001). The litter size in group 2 was significantly smaller than that in the control group (*P* < 0.05), and group 3 showed a dramatic decrease in litter size compared with the control group (*P* = 0.002). However, no significant difference was observed between the litter sizes of group 3 and group 4 (*P* > 0.05) (Figure 3).

### 3.3. Neonatal Death

**Figure 4:**
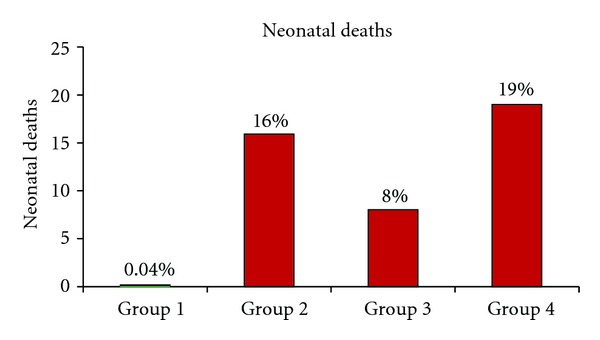
The percentages of neonatal deaths in the control group and in the three groups exposed to elevated temperature.

Neonatal deaths in all groups occurred within 24 h after parturition, mostly in the first 6 h. Neonatal death was increased in the groups exposed to elevated ambient temperatures (groups 2, 3, and 4) compared with the control group (Figure 4). The highest proportion of neonatal deaths was recorded in groups 2 and 4, while the lowest proportion was recorded in the control group.

### 3.4. Sex Ratio

**Figure 5:**
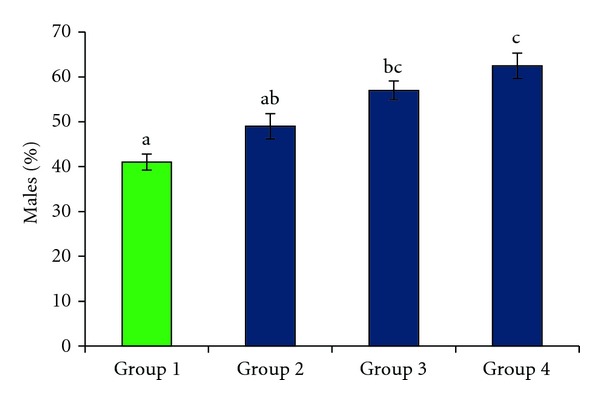
The percentages of males produced in the control group and in the three groups exposed to elevated temperature. Data represent the mean ± SEM. Data labeled with different letters are significantly different at *P* < 0.01.

More males were produced by the females of groups exposed to elevated ambient temperatures than those of the control group (Figure 5). Low and modest sex ratios were recorded in group 1 and group 2, respectively. The females in group 1 (control) produced offspring with 41% sex ratio, while the females in group 2 produced offspring with 49% sex ratio. By contrast, the sex ratios in group 3 and group 4 were biased toward males. The females in group 3 produced offspring with 57% sex ratio (*P* < 0.001), and the highest sex ratio was produced by the females in group 4, which delivered a significantly larger number of males (63%) than those of both group 1 (*P* < 0.001) and group 2 (*P* < 0.01). However, the sex ratios in groups 3 and 4 did not differ significantly (*P* > 0.05). Similarly, the sex ratios in group 1 (control) and group 2 were not significantly different (*P* > 0.05).

### 3.5. Growth Patterns

**Figure 6:**
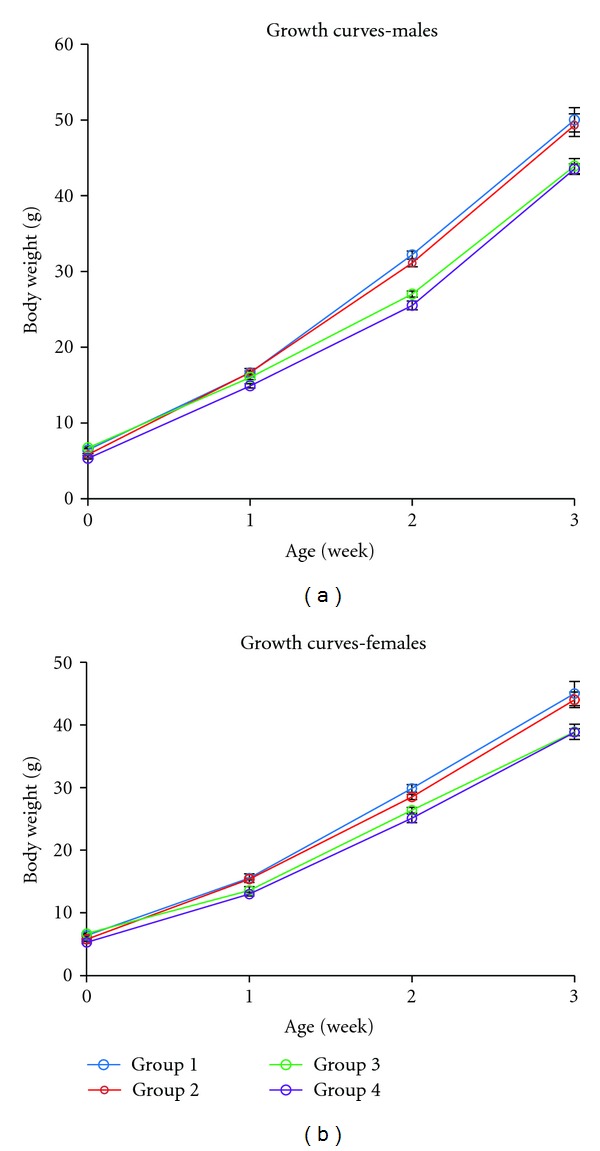
The growth curves for the male (a) and female (b) pups of the control group and the three groups exposed to elevated temperature from birth until weaning. Data represent the mean ± SEM.

The growth curves of the four groups are shown in Figure 6. No significant birth weight differences were observed between groups 1 and 3 and between groups 2 and 4 (*P* > 0.05). However, the group 2 pups showed significantly low birth weight compared with group 3 pups (*P* < 0.001).

The growth curves (Figures 6(a) and 6(b)) for both the male and female pups in group 1 (control) were significantly different from those of group 3 (*P* < 0.01) and group 4 (*P* < 0.001). No significant differences were observed between group 1 (control) and group 2 in the growth patterns of either the male or female pups (*P* > 0.05).

Surprisingly, the growth patterns of the group 3 and group 4 pups did not differ for either the males or females (*P* > 0.05). The growth pattern of the group 2 pups was significantly different from that of the group 3 pups (*P* < 0.01 for both males and females) and group 4 pups (*P* < 0.001 for both males and females).

## 4. Discussion

Maternal and paternal traits are strongly related to reproductive and productive efficiency. To control for parental factors, therefore, all of the rats used in this study were bred in our laboratory, and each of four littermate females were mated with the same male before being randomly assigned to one of the four groups. 

In mammals, gestation can be lengthened by delayed fertilization (sperm storage), delayed implantation (embryonic diapause), or delayed development [13]. In this study, an increase in gestation length was observed in groups 3 and 4. In group 3, the prolonged gestation period may have been due to an implantation delay because our previous study has shown that exposure to elevated temperatures during pre- and periimplantation can delay the implantation in rats [14]. Furthermore, the increased gestation time in group 4 may have been due to both delayed implantation and delayed development. This possibility is supported by a previous finding [13] that the fetal development may become slower in response to environmental cues, such as temperature. However, the gestation length of the group 2 rats was not significantly affected by the exposure to elevated temperatures during the developmental stages. This result suggests that rat embryos that undergo a delay in implantation are more susceptible to developmental delays than are normally implanted embryos.

Elevated ambient temperature during pregnancy decreased the litter size in all of the groups, but it appears that the harmful effect strongly depended on in which stage of pregnancy the exposure occurred. Although the litter sizes of the three groups exposed to elevated temperature were reduced, the litter size of group 2 would still be considered large (mean  =  9.8), suggesting that exposure to elevated temperature following implantation may not have a severe effect on litter size. By contrast, exposure to elevated temperature during pre- and periimplantation resulted in reduced litter size, as was noted in groups 3 and 4. Reduced litter size is likely a consequence of early embryonic loss because elevated temperature compromises embryonic survival during the developmental stages prior to the morula stage [15] and can severely decrease the implantation rates [14]. The lack of a significant difference between the litter sizes of groups 3 and 4 indicates that preimplantation exposure to elevated temperature may reduce litter size more strongly than postimplantation exposure. Nevertheless, fetal loss during the postimplantation period was demonstrated by the variation between the litter sizes of groups 1 and 2. 

Because the risk of neonatal death increases exponentially as birth weight decreases [16], the mortality of the neonatal rats in the exposed groups may be related to their low birth weights, with the highest rates of mortality being found among the group 2 and 4 pups. The high neonatal death rates in groups 2 and 4 confirm that the most detrimental effect of elevated ambient temperature on pup survival occurred when the pregnant rats were exposed to elevated temperature during the period from implantation to birth. In group 3, neonatal mortality among normal birth weight pups may be related to prolonged gestation period [17]. Therefore, the highest mortality rate in group 4 may have been due to both low birth weights and prolonged gestation. 

In this study, more males were produced by the three groups exposed to elevated ambient temperature. Skewed sex ratios have been a topic of great interest for more than a century. Many studies have been conducted to identify the causes associated with variations in sex ratios within and between populations. The causes of such changes are difficult to identify, although numerous demographic and environmental factors have been shown to be related to skewed sex ratios. These factors include maternal psychological stress, maternal diet, latitude, ambient temperature, and season [18–24]; however, the results concerning temperature are conflicting. 

Our finding of a male-skewed sex ratio following exposure to elevated temperature supports previous reports suggesting that a warm climate may increase sex ratios [19–21]. However, Navara [22] has reported that in countries at tropical latitudes, which have the highest average ambient temperatures, the sex ratios are lower than in countries at temperate and subarctic latitudes.

Variations in the sex ratio at birth are assumed to result from sex-biased fetal mortality [25], and increased mortality of the female fetuses in utero appears to have been responsible for the sex ratio bias toward males in this study. Although group 2 had a somewhat lower sex ratio, the proportion of males produced was still higher than in the control group. At the same time, the group 3 and 4 sex ratios were markedly biased toward males. The decrease in the proportion of females and reduced litter size in group 2 relative to the control group indicates that female fetuses were lost in the period between implantation and parturition because of exposure to elevated temperature.

It is clear that elevated ambient temperature can effectively increase the proportion of males, particularly if the exposure occurs during early pregnancy. The sex ratios in groups 2 and 3 confirm the findings of Evdokimova et al. [26], who observed that embryos aborted during the early stages of development are biased toward females (31% males), in contrast to late abortions (77% males).

The pups from groups 2 and 4 exhibited low birth weights. Surprisingly, the birth weight of pups from group 3 was not affected by prenatal heat exposure. The reduced birth weights of the group 2 and 4 pups may have been due to exposure to elevated ambient temperature during the developmental stages, which may have impaired the fetal growth [27]. 

In this study, no growth differences were observed between the pups of control and those of group 2 or between the pups of group 3 and those of group 4. The similarity in the growth patterns of the group 1 (control) and group 2 pups, regardless of their weight at birth, indicates the great importance of environmental conditions during early pregnancy for postnatal growth, at least in rats. This finding is confirmed by the similarity of the growth curves for the group 3 and 4 pups. 

No differences were observed between the male and female growth patterns in any of the groups; this result indicates that the implications of exposure to elevated ambient temperature have no relationship with the sex. 

In conclusion, elevated environmental temperature adversely affects reproductive performance. Exposure to elevated ambient temperature can lead to prolonged gestation time. This finding should be considered when making inferences about pregnancy due dates in the tropics and other regions with hot weather. Additionally, ambient temperature affects litter size, sex ratio, and the body weight. Exposure to elevated temperature during the early stages of pregnancy (the pre- and periimplantation stages) has stronger adverse effects on pregnancy outcomes and offspring growth than does exposure during the mid and late stages of pregnancy.
